# Supplementary material for: Genome-Wide Identification and Characterization of the CCT Gene Family in Foxtail Millet (Setaria italica) Response to Diurnal Rhythm and Abiotic Stress
Source: Genes (Basel). 2022 Oct 10;13(10):1829. doi: 10.3390/genes13101829 (PMC9601966; doi:10.3390/genes13101829)
Supplement: Supplementary file 1 [file genes-13-01829-s001.zip › Supplementary Figures.pdf]

Genome-wide identification and characterization of the *CCT* gene family in foxtail millet (*Setaria italica*) response to diurnal rhythm and abiotic stress

Yuntong Li[1†], Shumin Yu[1†], Qiyuan Zhang[1], Ziwei Wang[1], Meiling Liu[1], Ao Zhang[1], Xiaomei Dong[1], Jinjuan Fan[1], Yanshu Zhu[1], Yanye Ruan[1], and Cong Li[1]*

[1] College of Bioscience and Biotechnology, Shenyang Agricultural University, Shenyang, China

* Correspondence: lnlicong_123@syau.edu.cn

† These authors contributed equally to this work.

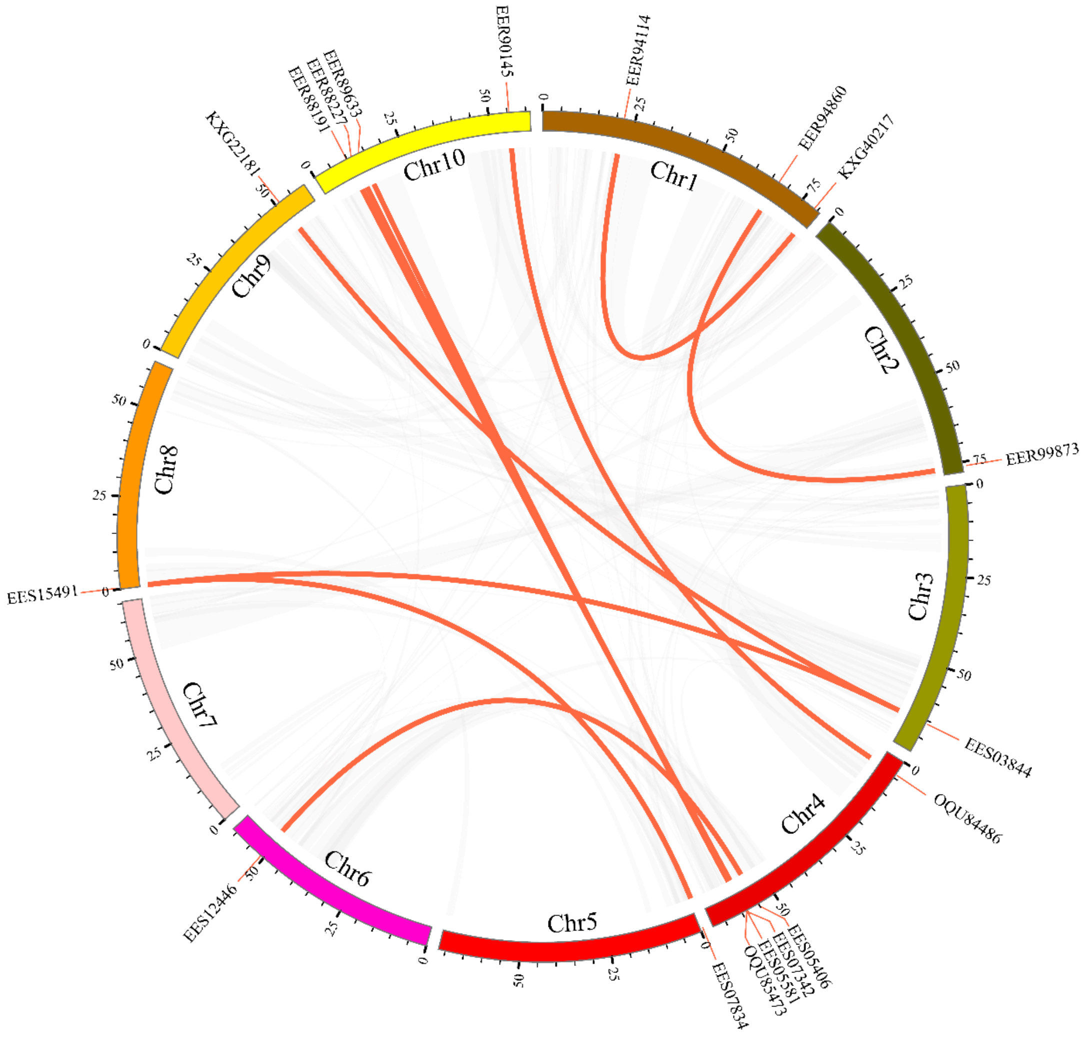

Figure S1. Interchromosomal relationships of sorghum CCT genes.

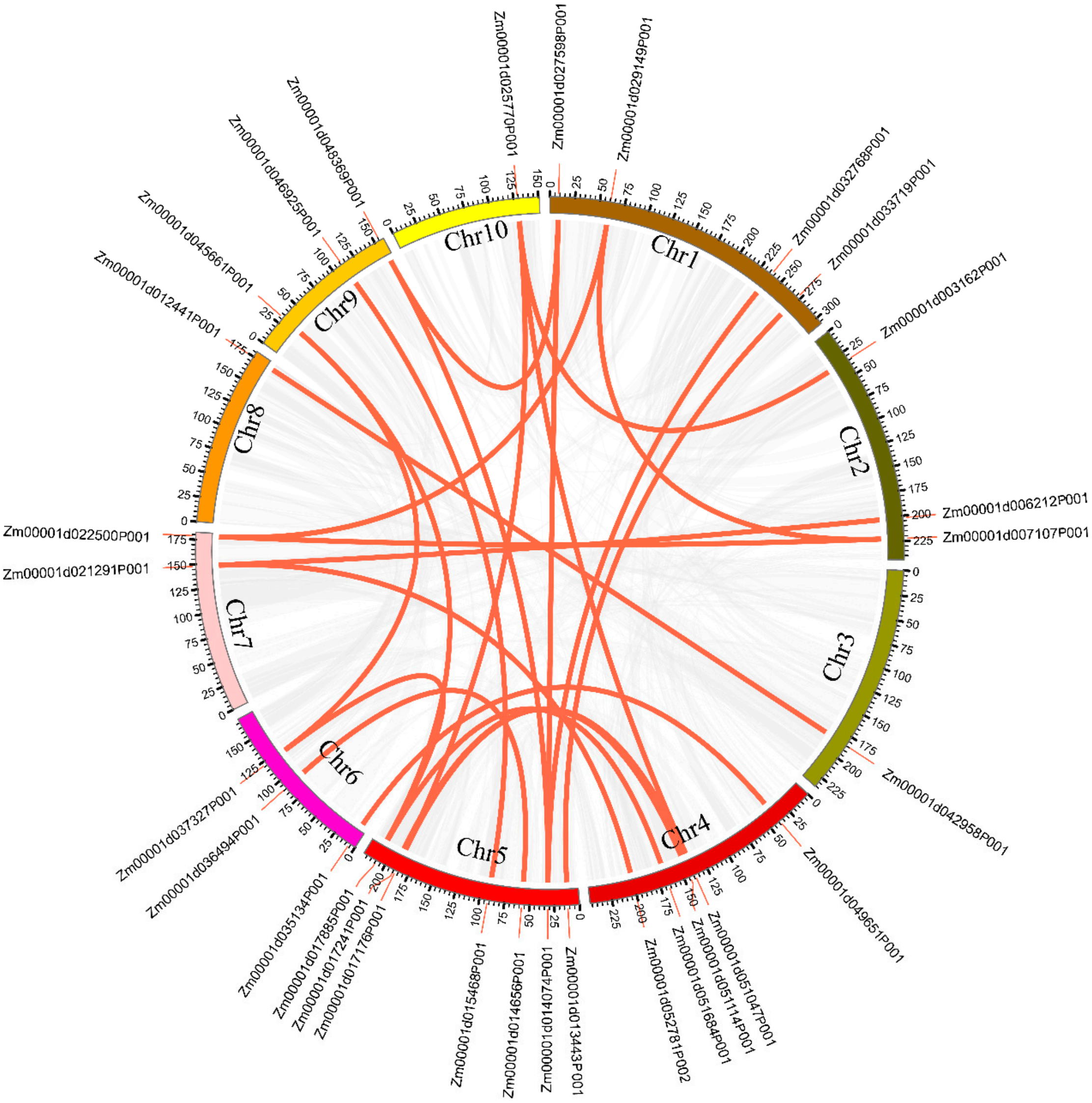

Figure S2. Interchromosomal relationships of maize CCT genes.

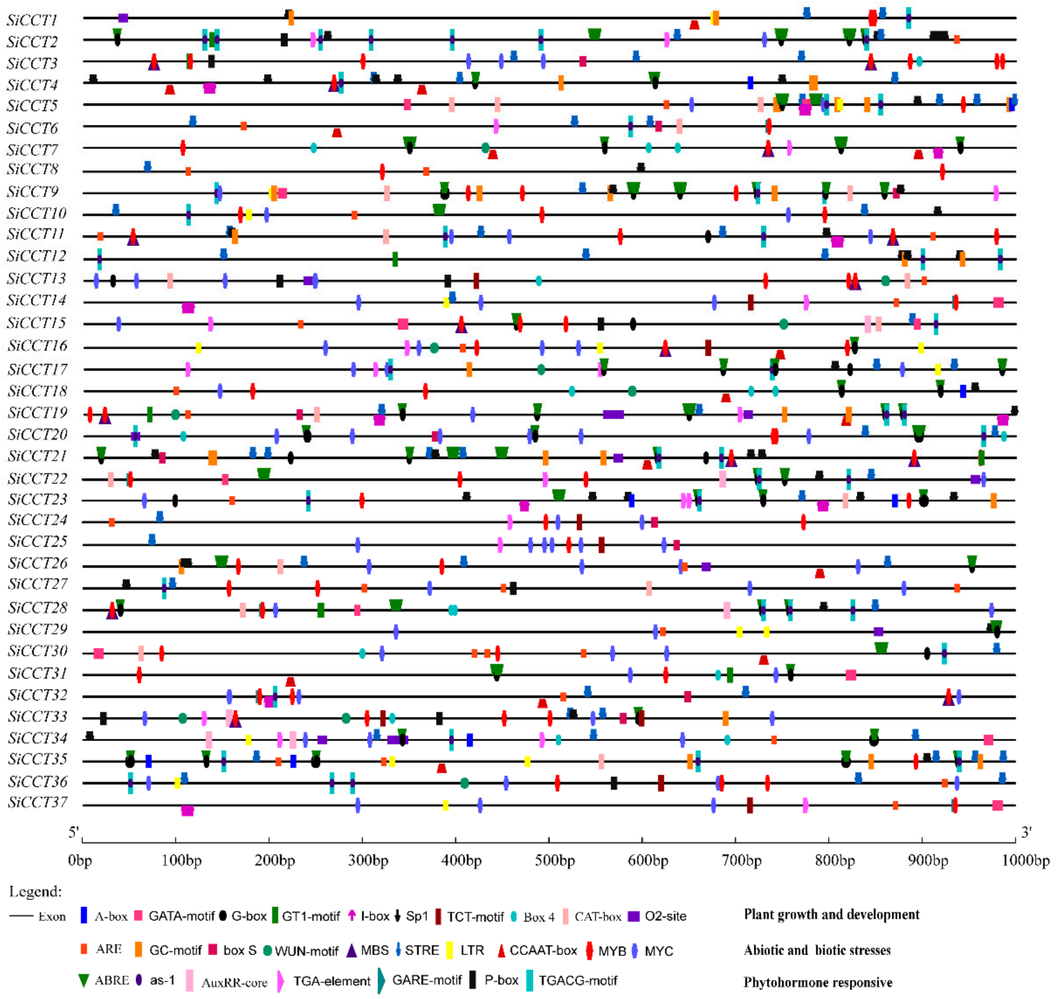

Figure S3. Distribution map of cis-acting elements on each gene.

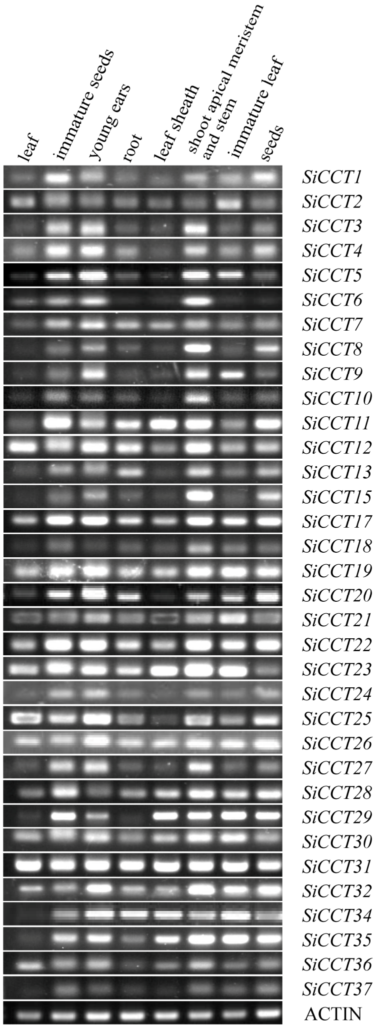

Figure S4. Expression profiles of SiCCT genes in different tissues of millet. The site of expression is shown at the top, and the gene name is shown on the right.

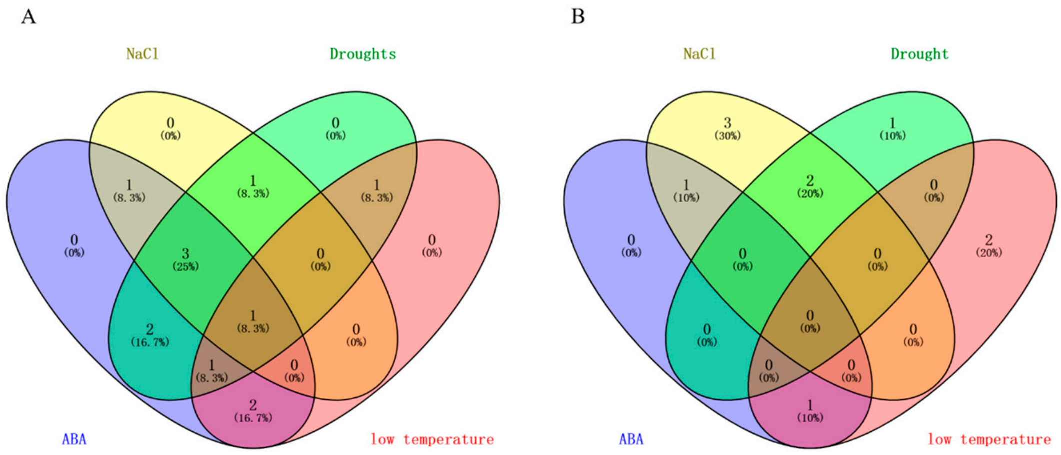

Figure S5. The detailed numbers of simultaneously up-regulated (**A**) and down-regulated (**B**) *SiCCT* genes by ABA (abscisic acid), NaCl, drought, and low temperature.
