# Supplementary material for: Genome-Wide Identification and Characterization of the CCT Gene Family in Foxtail Millet (Setaria italica) Response to Diurnal Rhythm and Abiotic Stress
Source: Genes (Basel). 2022 Oct 10;13(10):1829. doi: 10.3390/genes13101829 (PMC9601966; doi:10.3390/genes13101829)
Supplement: Supplementary file 1 [file genes-13-01829-s001.zip › Table S1.pdf]

Table S1 Primer name and sequence information of *SiCCT*

Primer name	Primer sequences	Number of bases
SiqCCT-1-1-F	5'--TCAGGATGCGGTGGCTCTTCA--3'	21
SiqCCT-1-1-R	5'--GCCTCTTCTCTTGCTGCTTCGT--3'	22
SiqCCT-2-1-F	5'--CTACCCACAGTGTGTCGTCTTTGAA--3'	25
SiqCCT-2-1-R	5'--ACCGCCTGTTCTTCCGCTTCT--3'	21
SiqCCT-3-1-F	5'--CGCCAGAGAAGAAGAAGAGCAAGA--3'	24
SiqCCT-3-1-R	5'--TTGTCGGACCACGCCTTGAG--3'	20
SiqCCT-4-1-F	5'--AATCCTTCTGTTGCTGAGCCTGAG--3'	24
SiqCCT-4-1-R	5'--GAATCTAACCGACTGCCACCTCTT--3'	24
SiqCCT-5-1-F	5'--TGGCATCCTCCTGGTCCTGAAG--3'	22
SiqCCT-5-1-R	5'--TTGGCTTAGCGGATCGTAGTCATAC--3'	25
SiqCCT-6-1-F	5'--AGTAGACTCCTGGCTCCTGTTCAC--3'	24
SiqCCT-6-1-R	5'--CACTCCATTCCGTTGCTCCATACC--3'	24
SiqCCT-7-1-F	5'--GGAGCCTGGATCTCAGACTCAACT--3'	24
SiqCCT-7-1-R	5'--CGCACCTCGTACCGTATCTTCTTG--3'	24
SiqCCT-8-1-F	5'--CAATGCGGTATCTGCTGACTCTGG--3'	24
SiqCCT-8-1-R	5'--AGACTGGACCTAGCCTGCCTTG--3'	22
SiqCCT-9-1-F	5'--TCTGCCATAAGTCCTCCTAATTCCA--3'	25
SiqCCT-9-1-R	5'--CCGCTGCCGCTGAGAGTATT--3'	20
SiqCCT-10-1-F	5'--GGCTTCATCAGAGTATGGCATTGGA--3'	25
SiqCCT-10-1-R	5'--GTTGCTGTCGTCGGTCGTCATC--3'	22
SiqCCT-11-1-F	5'--CAATGACAACGACGAGGACGATGA--3'	24
SiqCCT-11-1-R	5'--TCTGGAGACATAGCCTGTGGACTG--3'	24
SiqCCT-12-1-F	5'--AACGAACCTTCAGACGCCTTGTG--3'	23
SiqCCT-12-1-R	5'--TCAGCAGGTCGCATTCTTCAATCTT--3'	25
SiqCCT-13-1-F	5'--CACCTTCTCTTGCTCGTCTCCTTC--3'	24
SiqCCT-13-1-R	5'--GCTCAATCTTCTCCTTGCGTTCTTC--3'	25
SiqCCT-15-1-F	5'--CGCTTGTATATAAAGAAACGGAACG--3'	25
SiqCCT-15-1-R	5'--GCAAACATTCCACGGACACG--3'	20
SiqCCT-17-1-F	5'--GGTGCCATCGAGCTGGACTTCA--3'	22
SiqCCT-17-1-R	5'--CGACGACACGCTGTGACTCTGA--3'	22
SiqCCT-18-1-F	5'--AGCAGCAGCAGCAGCAGATG--3'	20
SiqCCT-18-1-R	5'--CTCTTCTTCCTCGCCGTCCTCT--3'	22
SiqCCT-19-1-F	5'--GCTGCTGCTCAAGCTGGACTAC--3'	22
SiqCCT-19-1-R	5'--TTCTCCTTGTATCGCCTCACCTTCT--3'	25
SiqCCT-20-1-F	5'--AACCGCAAGTTCCAGAAGACCATC--3'	24
SiqCCT-20-1-R	5'--CGTCGGAGAAGGAGAGCCAGAA--3'	22
SiqCCT-21-1-F	5'--ACGATGGATGGACCGACAACCT--3'	22
SiqCCT-21-1-R	5'--GGATGTGCTTCTCATACCTGCGATT--3'	25
SiqCCT-22-1-F	5'--CGAGCCGCCTTCTCCATCTTCA--3'	22
SiqCCT-22-1-R	5'--GATGCTTTCGTGGTCGTCCTGTC--3'	23
SiqCCT-23-1-F	5'--ACAGCGTGTCATCGTCGGAAG--3'	21
SiqCCT-23-1-R	5'--ACATCTCCTCCTCGTGCTCCTC--3'	22

SiqCCT-24-1-F	5'--ATTCCAATGCCATCCTCTCCTATCG--3'	25
SiqCCT-24-1-R	5'--TACCGTTGCTCTTCCTCCCTGTC--3'	23
SiqCCT-25-1-F	5'--CGGGAGCGCATCGACAAGTA--3'	20
SiqCCT-25-1-R	5'--AGAGTCTTCCGGCAAGCATACG--3'	22
SiqCCT-26-1-F	5'--GAAGTGAGAAGAATGTGCGTTCCAA--3'	25
SiqCCT-26-1-R	5'--TCAGCAATCCTCAAGGCATTATCCA--3'	25
SiqCCT-27-1-F	5'--TCCTCATGCTTCTAAACGGCTACGA--3'	25
SiqCCT-27-1-R	5'--GCTGCTGCGGCGACTGAATT--3'	20
SiqCCT-28-1-F	5'--TGAAAGCGTTGCCTGGCTCTTC--3'	22
SiqCCT-28-1-R	5'--TTGGTGAGGCTGGTTCTCAACTG--3'	23
SiqCCT-29-1-F	5'--TGGTGATGGTGCGTGTTCTTCTG--3'	23
SiqCCT-29-1-R	5'--GGTAAGTCTTGAGCTGATGCCACAA--3'	25
SiqCCT-30-1-F	5'--GCCTCAGGTGAACGTGGACTTG--3'	22
SiqCCT-30-1-R	5'--GCCGCTTCCTCTTCTTGTCATCTC--3'	24
SiqCCT-31-1-F	5'--GCTCCTGGCTGTGATCCTGGTT--3'	22
SiqCCT-31-1-R	5'--TGTGGCTTCGCTGTTGCTTGC--3'	21
SiqCCT-32-1-F	5'--ACTCCACCTTATCGGCTCTCCTC--3'	23
SiqCCT-32-1-R	5'--TGCTGCTCATCTGCGTCTGC--3'	20
SiqCCT-33-1-F	5'--AGAGCGTGACGTGGCTGTTC--3'	20
SiqCCT-33-1-R	5'--GCTCCCGCAGAAGGACATGATTT--3'	23
SiqCCT-34-1-F	5'--CTGCGTCTCATCCTCTTGCTCTCA--3'	24
SiqCCT-34-1-R	5'--CCTGCTTCCTCTGGTCTTCCTCAC--3'	24
SiqCCT-35-1-F	5'--TGCTCCGTAAGTGCTGCTATGAAG--3'	24
SiqCCT-35-1-R	5'--CAGACCGATGCCAGATAGACAAGG--3'	24
SiqCCT-36-1-F	5'--CAGCAAGCAGGAGCAATTCTTACG--3'	24
SiqCCT-36-1-R	5'--TCTCTTCAGGTGACAATCGGACAAC--3'	25
SiqCCT-37-1-F	5'--TGGATGGAGCCATGTACCAGCAA--3'	23
SiqCCT-37-1-R	5'--TTCTTCAGGTGACAAGCGGACAAC--3'	24
Actin-F	5'--GTGCTTTCCCTCTACGCCAGTG--3'	22
Actin-R	5'--ACCGCTGAGCACAATGTTACCA--3'	22

Actin gene (*SETIT_010361mg*) was used as internal control for this experiment.
