# Supplementary material for: Genome-Wide Identification and Characterization of the CCT Gene Family in Foxtail Millet (Setaria italica) Response to Diurnal Rhythm and Abiotic Stress
Source: Genes (Basel). 2022 Oct 10;13(10):1829. doi: 10.3390/genes13101829 (PMC9601966; doi:10.3390/genes13101829)
Supplement: Supplementary file 1 [file genes-13-01829-s001.zip › Table S2.pdf]

Table S2: List of *CCT* genes in *Arabidopsis*, rice, and foxtail millet.

Plant Specie	Transcript ID	Gene Name	Gene Symbol	Gene Family
Setaria italica	KQL27760	*SETIT_019213mg*	*SiCCT1*	COL
Setaria italica	KQL28473	*SETIT_017487mg*	*SiCCT2*	COL
Setaria italica	KQL31286	*SETIT_017374mg*	*SiCCT5*	COL
Setaria italica	KQL31326	*SETIT_019803mg*	*SiCCT6*	COL
Setaria italica	KQL31374	*SETIT_017124mg*	*SiCCT7*	COL
Setaria italica	KQL24119	*SETIT_030034mg*	*SiCCT8*	COL
Setaria italica	KQL27073	*SETIT_030140mg*	*SiCCT10*	COL
Setaria italica	KQL10228	*SETIT_006432mg*	*SiCCT16*	COL
Setaria italica	KQL11132	*SETIT_006690mg*	*SiCCT17*	COL
Setaria italica	KQL01176	*SETIT_014224mg*	*SiCCT20*	COL
Setaria italica	KQK97912	*SETIT_010592mg*	*SiCCT23*	COL
Setaria italica	KQK87465	*SETIT_035937mg*	*SiCCT30*	COL
Setaria italica	KQK90887	*SETIT_034611mg*	*SiCCT34*	COL
Arabidopsis thaliana	AT5G57660.1	*AT5G57660*	COL5	COL
Arabidopsis thaliana	AT2G24790.1	*AT2G24790*	COL3	COL
Arabidopsis thaliana	AT5G15850.1	*AT5G15850*	COL1	COL
Arabidopsis thaliana	AT3G07650.1	*AT3G07650*	COL9	COL
Arabidopsis thaliana	AT5G24930.1	*AT5G24930*	COL4	COL
Arabidopsis thaliana	AT4G15250.1	*AT4G15250*	COL11	COL
Arabidopsis thaliana	AT1G68520.1	*AT1G68520*	COL6	COL
Arabidopsis thaliana	AT1G73870.1	*AT1G73870*	COL7	COL
Arabidopsis thaliana	AT5G48250.1	*AT5G48250*	COL10	COL
Arabidopsis thaliana	AT1G25440.1	*AT1G25440*	COL16	COL
Arabidopsis thaliana	AT5G15840.1	*AT5G15840*	CO	COL
Arabidopsis thaliana	AT2G47890.1	*AT2G47890*	COL13	COL
Arabidopsis thaliana	AT2G33500.1	*AT2G33500*	COL14	COL
Arabidopsis thaliana	AT1G49130.1	*AT1G49130*		COL
Arabidopsis thaliana	AT3G02380.1	*AT3G02380*	COL2	COL
Arabidopsis thaliana	AT1G28050.1	*AT1G28050*	COL15	COL
Arabidopsis thaliana	AT3G21880.1	*AT3G21880*		COL
Oryza sativa Japonica	Os02t0724000-01	*Os02g0724000*	DTH2	COL
Oryza sativa Japonica	Os06t0264200-00	*Os06g0264200*		COL
Oryza sativa Japonica	Os09t0509700-01	*Os09g0509700*		COL
Oryza sativa Japonica	Os04t0497700-01	*Os04g0497700*		COL
Oryza sativa Japonica	Os06t0275000-01	*Os06g0275000*	Hd1	COL
Oryza sativa Japonica	Os08t0536300-01	*Os08g0536300*		COL
Oryza sativa Japonica	Os07t0667300-01	*Os07g0667300*		COL
Oryza sativa Japonica	Os03t0711100-01	*Os03g0711100*		COL
Oryza sativa Japonica	Os03t0351100-01	*Os03g0351100*		COL
Oryza sativa Japonica	Os06t0298200-01	*Os06g0298200*		COL
Oryza sativa Japonica	Os02t0610500-01	*Os02g0610500*		COL
Oryza sativa Japonica	Os02t0178100-01	*Os02g0178100*		COL
Oryza sativa Japonica	Os09t0240200-01	*Os09g0240200*		COL

Plant Specie	Transcript ID	Gene Name	Gene_Symbol	Gene Family
Setaria italica	KQL28750	*SETIT_017125mg*	*SiCCT3*	CMF
Setaria italica	KQL13967	*SETIT_022659mg*	*SiCCT12*	CMF
Setaria italica	KQL15112	*SETIT_024852mg*	*SiCCT13*	CMF
Setaria italica	KQL16011	*SETIT_024038mg*	*SiCCT14*	CMF
Setaria italica	KQL16012	*SETIT_024806mg*	*SiCCT15*	CMF
Setaria italica	KQL11783	*SETIT_006750mg*	*SiCCT18*	CMF
Setaria italica	KQL01011	*SETIT_014122mg*	*SiCCT19*	CMF
Setaria italica	KQL02690	*SETIT_014037mg*	*SiCCT21*	CMF
Setaria italica	KQK96183	*SETIT_011920mg*	*SiCCT22*	CMF
Setaria italica	KQL00040	*SETIT_011862mg*	*SiCCT24*	CMF
Setaria italica	KQK93230	*SETIT_027626mg*	*SiCCT25*	CMF
Setaria italica	KQK95022	*SETIT_027518mg*	*SiCCT27*	CMF
Setaria italica	KQK86275	*SETIT_039184mg*	*SiCCT28*	CMF
Setaria italica	KQK87242	*SETIT_036747mg*	*SiCCT29*	CMF
Setaria italica	KQK87704	*SETIT_036492mg*	*SiCCT31*	CMF
Setaria italica	KQK88797	*SETIT_035901mg*	*SiCCT32*	CMF
Setaria italica	KQK89929	*SETIT_036910mg*	*SiCCT33*	CMF
Setaria italica	KQK92647	*SETIT_039219mg*	*SiCCT36*	CMF
Setaria italica	KQK85264	*SETIT_020907mg*	*SiCCT37*	CMF
Arabidopsis thaliana	AT5G41380.1	*AT5G41380*		CMF
Arabidopsis thaliana	AT4G27900.1	*AT4G27900*		CMF
Arabidopsis thaliana	AT2G33350.1	*AT2G33350*		CMF
Arabidopsis thaliana	AT4G25990.1	*AT4G25990*	*CIL*	CMF
Arabidopsis thaliana	AT1G04500.1	*AT1G04500*		CMF
Arabidopsis thaliana	AT5G14370.1	*AT5G14370*		CMF
Arabidopsis thaliana	AT5G53420.1	*AT5G53420*		CMF
Arabidopsis thaliana	AT1G63820.1	*AT1G63820*		CMF
Arabidopsis thaliana	AT1G07050.1	*AT1G07050*		CMF
Arabidopsis thaliana	AT5G59990.1	*AT5G59990*		CMF
Oryza sativa Japonica	Os05t0466100-01	*Os05g0466100*		CMF
Oryza sativa Japonica	Os03t0139500-01	*Os03g0139500*		CMF
Oryza sativa Japonica	Os05t0595300-02	*Os05g0595300*		CMF
Oryza sativa Japonica	Os02t0148000-01	*Os02g0148000*		CMF
Oryza sativa Japonica	Os10t0560400-01	*Os10g0560400*		CMF
Oryza sativa Japonica	Os11t0101200-01	*Os11g0101200*		CMF
Oryza sativa Japonica	Os07t0261200-01	*Os07g0261200*	*GHD7*	CMF
Oryza sativa Japonica	Os06t0699600-01	*Os06g0699600*		CMF
Oryza sativa Japonica	Os01t0835700-01	*Os01g0835700*		CMF
Oryza sativa Japonica	Os12t0101000-01	*Os12g0101000*		CMF
Oryza sativa Japonica	Os08t0119700-00	*Os08g0119700*		CMF
Oryza sativa Japonica	Os10t0466500-01	*Os10g0466500*		CMF

Plant Specie	Transcript ID	Gene Name	gene_symbol	Gene Family
Setaria italica	KQL30521	*SETIT_016922mg*	*SiCCT4*	PRR
Setaria italica	KQL25444	*SETIT_029202mg*	*SiCCT9*	PRR
Setaria italica	KQL27318	*SETIT_033274mg*	*SiCCT11*	PRR
Setaria italica	KQK91381	*SETIT_034368mg*	*SiCCT35*	PRR
Setaria italica	KQK93667	*SETIT_026170mg*	*SiCCT26*	PRR
Arabidopsis thaliana	AT2G46790.1	*AT2G46790*	*APRR9*	PRR
Arabidopsis thaliana	AT5G60100.1	*AT5G60100*	*PRR3*	PRR
Arabidopsis thaliana	AT5G61380.1	*AT5G61380*	*APRR1*	PRR
Arabidopsis thaliana	AT5G24470.1	*AT5G24470*	*APRR5*	PRR
Arabidopsis thaliana	AT5G02810.1	*AT5G02810*	*APRR7*	PRR
Oryza sativa Japonica	Os09t0532400-01	*Os09g0532400*		PRR
Oryza sativa Japonica	Os02t0618200-01	*Os02g0618200*		PRR
Oryza sativa Japonica	Os11t0157600-01	*Os11g0157600*		PRR
Oryza sativa Japonica	Os07t0695100-01	*Os07g0695100*	*Hd2*	PRR
Oryza sativa Japonica	Os03t0284100-01	*Os03g0284100*		PRR
